# Mechanisms of moral disengagement as predictors of direct and indirect bullying perpetration: a short-term longitudinal study

**DOI:** 10.1186/s40359-026-05156-3

**Published:** 2026-07-15

**Authors:** Björn Sjögren, Robert Thornberg, Jun Sung Hong, Mattias Kloo

**Affiliations:** 1 Department of Behavioral Sciences and Learning, Linköping University, Linköping, 58183 Sweden; 2 School of Social Work, Wayne State University, Detroit, USA; 3 Department of Social Welfare, Ewha Womans University, Seoul, South Korea; 4 INVEST Research Flagship, Department of Psychology, University of Turku, Turku, Finland

**Keywords:** Moral disengagement, Bullying, Social cognitive theory

## Abstract

The current study explored whether specific mechanisms of moral disengagement predicted bullying perpetration from a sample of upper elementary school students in Sweden. The study hypothesized that both moral justification (portraying harmful actions as serving a noble or socially valuable purpose) and victim attribution (blaming the victims for their own suffering) would be positively associated with subsequent bullying perpetration. Participants, who consisted of 471 students in grades 4–6, completed a web-based questionnaire at two timepoints. Analyses included two linear regression analyses—one with indirect bullying and one with direct bullying. Results indicated that moral justification significantly predicted bullying. The victim attribution predicted higher levels of indirect bullying and was significantly associated with bullying among girls. The findings suggest that assessing students’ morals and identifying and intervening in disengagement patterns might potentially be avenues practitioners need to consider. Also, programs need to consider gender differences and specifically target victim-blaming attitudes.

## Introduction

Bullying is a pervasive social issue that affects children and adolescents across diverse contexts and cultures. Defined by repeated aggressive behaviors characterized by an imbalance of power [17], bullying can manifest in both direct forms, such as physical and verbal aggression, and indirect forms, such as exclusion or spreading of rumors [36]. Over the past two decades, research has increasingly emphasized the significance of examining moral cognition to better understand why children and adolescents engage in bullying behaviors. Specifically, the concept of moral disengagement has been highlighted as a crucial factor in this context. A meta-analysis by Killer et al. [18] highlights the strong relationship between moral disengagement and bullying roles in youth. This suggests that moral disengagement may help explain why some individuals nonetheless engage in bullying, despite recognizing it as morally wrong due to its harmful nature [3, 16, 30].

### Moral disengagement

Moral disengagement refers to social-cognitive processes by which individuals justify or rationalize harmful or unethical behavior, thereby alleviating feelings of guilt or self-condemnation that would typically follow such actions [2]. Introduced within Bandura's social cognitive theory, the concept of moral disengagement explains how individuals who generally distinguish right from wrong may nonetheless engage in behaviors inconsistent with their moral standards. Central to this concept is the notion that moral standards are internalized through socialization processes; however, individuals may disengage these standards under certain circumstances, enabling morally questionable actions without experiencing negative self-sanctions such as guilt or remorse [1, 2].

Bandura [2] identifies eight distinct mechanisms through which moral disengagement operates, organized into four loci: behavioral, agency, effects, and victim. The behavioral locus involves processes that reframe harmful behaviors as acceptable or justified, including moral justification (portraying harmful actions as serving a noble or socially valuable purpose), euphemistic labeling (using language that renders harmful acts appear less severe), and advantageous comparison (comparing negative behaviors with more egregious acts to diminish their perceived severity). The agency locus encompasses mechanisms by which individuals minimize their accountability, including displacement of responsibility (attributing responsibility to authorities or higher-status figures) and diffusion of responsibility (distributing responsibility across a group). The effect locus pertains to the cognitive distortion or minimization of consequences, whereby individuals minimize or disregard the harm caused by their actions. Finally, the victim locus includes two mechanisms: dehumanization (denying or diminishing the humanity of victims) and victim attribution (blaming the victims for their own suffering) [1, 2].

### Moral disengagement and bullying perpetration

Previous research underscores the role of moral disengagement as a predictor of both traditional bullying behaviors and cyberbullying perpetration among children and adolescents [18, 37]. Moral disengagement may help explain why some individuals nonetheless engage in bullying, despite recognizing it as morally wrong due to its harmful nature [9]. From a developmental perspective, these cognitive processes may create a reinforcing cycle: engaging in bullying can prompt individuals to increasingly employ moral disengagement mechanisms, thereby justifying future bullying behavior. Conversely, the ongoing activation of these mechanisms facilitates further involvement in bullying by reducing feelings of guilt and remorse, thus reinforcing a reciprocal relationship over time (see also [32].

In their meta-analysis, Gini et al. [16] observed that much of the previous literature investigating moral disengagement and aggressive behaviors, including bullying, has treated moral disengagement as a unidimensional construct, despite Bandura’s [2] conceptualization of it as multidimensional. Examining the distinct mechanisms may provide more precise and practically relevant knowledge for school-based prevention programs to target youth’s cognitive justifications in bullying.

However, only a limited number of studies have examined how distinct moral disengagement mechanisms are separately related to bullying perpetration. For example, Robson and Witenberg [24] found that bullying perpetration among Australian students aged 12–15 years was associated with the two mechanisms of moral justification and diffusion of responsibility. Similarly, Thornberg and Jungert [30], in a study of Swedish early adolescents aged 10–14 years, found that moral justification was positively associated with bullying perpetration. However, in contrast to Robson and Witenberg [24], diffusion of responsibility was not found to be associated with bullying perpetration. Instead, they identified victim attribution as a significant correlate. Expanding upon these findings, Bjärehed et al. [3] further investigated how specific moral disengagement mechanisms relate to direct and indirect bullying perpetration, as well as to pro-aggressive bystander behavior, among Swedish students aged 10–15 years. Their study revealed that moral justification was consistently associated with direct bullying, aligning with previous findings [24, 30]. Additionally, victim attribution emerged as significantly linked to both direct and indirect bullying, highlighting its particular relevance across bullying types—a finding consistent with Thornberg and Jungert [30]. Importantly, Bjärehed et al. [3] also underscored the role of diffusion of responsibility, though this mechanism was specifically associated with pro-aggressive bystander behavior rather than with bullying perpetration as in Robson & Witenberg [24]. Taken together, these findings emphasize that moral disengagement mechanisms may operate differently depending on the specific form of bullying or the particular role an individual assumes in bullying situations.

While the studies reviewed above have provided important insights into the specific moral disengagement mechanisms associated with aggression and bullying perpetration, their cross-sectional designs preclude conclusions regarding temporal order. This limitation is significant because it prevents the establishment of causal relationships, making it difficult to determine whether moral disengagement mechanisms precede and influence bullying behaviors or vice versa. However, only a few studies have employed a longitudinal design to investigate whether specific mechanisms of moral disengagement predict future bullying. In two studies, Falla et al. [11, 12] identified positive longitudinal bivariate correlations: behavioral locus, agency locus, effect locus, and dehumanization mechanism correlated with later bullying perpetration among Spanish adolescents. However, only Falla et al. [12] examined these loci and dehumanization concurrently within the same multivariate model (controlling for age, gender, and previous peer victimization), revealing that only the behavioral locus significantly predicted bullying perpetration six months later. Consistent with this, Marín-López et al. [19] found that moral justification predicted cyberbullying, rather than traditional bullying as investigated in Falla et al. [11, 12] perpetration one year later, although their focus was limited to this single mechanism rather than the broader behavioral locus of which it is a part.

This gap in longitudinal research limits our understanding of the extent to which specific moral disengagement mechanisms predict future bullying perpetration among children and adolescents. Furthermore, examining direct and indirect forms of bullying separately—which previous longitudinal studies, such as Falla et al. [12], have not done—could further clarify whether particular moral disengagement mechanisms are uniquely predictive of specific bullying behaviors. Clarifying these predictive relationships is important both theoretically and practically, as it could inform targeted interventions designed to disrupt maladaptive social-cognitive patterns and prevent direct and indirect bullying behaviors from becoming deeply entrenched. More specifically, understanding which specific moral disengagement mechanisms are uniquely predictive of particular bullying behaviors allows for the development of more precise and effective interventions. This precision is crucial for tailoring interventions to address the specific cognitive processes that underlie different forms of bullying, potentially enhancing their effectiveness. Additionally, recognizing that different schools, classes, and contexts may have varying degrees of prevalence and issues with different types of bullying can further inform the design of context-specific interventions.

### The current study

The aim of the present study was to investigate whether specific mechanisms of moral disengagement predict subsequent bullying perpetration among Swedish upper elementary school students across two measurement points within the same academic year. The focus on students in upper elementary school (grades 4–6) is especially relevant, as previous research has demonstrated that bullying behaviors tend to peak among children in this age group, both in Sweden (Friends 14 and internationally [6]. Moreover, this developmental period is characterized by substantial and rapid changes across multiple domains, including cognitive, emotional, social, and physical growth [29]. It is also a critical stage during which children increasingly develop their individual identities, seek greater autonomy, and become more strongly influenced by their peer groups [4].

Drawing on prior cross-sectional findings that identified moral justification and victim attribution as particularly salient mechanisms of moral disengagement in bullying perpetration,even when controlling for other mechanisms and covariates [3, 24, 30]. Additionally, limited longitudinal evidence suggests that the behavioral locus [12], and specifically the mechanism of moral justification within it [19], predicts later bullying behavior in multivariate models. Based on these findings, we hypothesized that both moral justification and victim attribution would be positively associated with subsequent bullying perpetration. Beyond these mechanisms, previous studies have also consistently shown that all moral disengagement mechanisms tend to exhibit significant bivariate correlations, including longitudinal correlations [11], with bullying perpetration. Therefore, we also examined the full set of mechanisms as potential longitudinal predictors of both direct and indirect bullying perpetration. Additionally, gender was included as a control variable, as previous research indicates associations between gender and bullying perpetration, as well as between gender and moral disengagement mechanisms (e.g., [24, 30]. We also tested interactions between gender and moral disengagement mechanisms to explore whether there were gender differences in how these mechanisms predict direct and indirect bullying, an approach also taken by Bjärehed et al. [3], who found significant interaction between gender and victim attribution on direct bullying.

## Method

### Participants

Participants completed a web-based questionnaire at two time points within the same academic year. Participation varied across time points, with 578 students responding at Time 1 and 523 at Time 2. Of these, 471 students (55% girls, age range = 9.9–12.8, *M* = 11.8 years) provided sufficient data—defined as complete responses on gender, moral disengagement variables at Time 1, and bullying perpetration at Time 1 and Time 2—to be included in the longitudinal regression analyses. These students were enrolled in grade 4 (*n* = 30), grade 5 (*n* = 241), and grade 6 (*n* = 200). To assess potential attrition effects, we compared students included in the longitudinal analyses with those who were excluded, based on their levels of moral disengagement and bullying perpetration at Time 1. Attrition effects refer to the loss of participants over time, which can potentially bias the study results by affecting the representativeness of the sample. By comparing the included and excluded students, we aimed to evaluate whether the loss of participants had a significant impact on the study outcomes. Independent *t*-tests revealed no significant differences at Time 1 in any of the moral disengagement mechanisms or bullying perpetration between students who were included in the longitudinal analyses and those excluded due to missing data on bullying perpetration at Time 2: moral justification *p* = 0.85; euphemistic labeling *p* = 0.83; advantageous comparison *p* = 0.17; displacement of responsibility *p* = 0.58; diffusion of responsibility *p* = 0.36; distorting consequences *p* = 0.77; victim attribution *p* = 0.49; indirect bullying *p* = 0.91; direct bullying *p* = 0.29. The sample comprised students from a variety of socioeconomic backgrounds, ranging from lower to upper-middle class, and from diverse socio-geographic settings, including rural areas as well as medium-sized and large urban municipalities. Data were collected across eight different municipalities. In total, 13% of the students had an immigrant background, defined as either being born outside of Sweden or having two parents born abroad.

### Procedure

Before the commencement of the study, ethical approval was obtained from the Swedish Ethical Review Authority. School principals and teachers were informed about the project and provided their approval for data collection in classrooms. Written informed consent was obtained from parents, and all participating students gave their assent. Data were gathered through a web-based questionnaire administered on tablets during school hours, at two measurement points within a single school year: the first in November–December (w1) and the second in February–March (w2). Students received standardized instructions before beginning the survey and were informed that participation was voluntary and confidential. They were also made aware of their right to withdraw from the study at any time without providing a reason. Completion of the questionnaire typically required 20–30 min. As all variables in this study were self-reported, there is a risk of common method variance [23], which may inflate the observed associations between moral disengagement and bullying perpetration. While we took steps to ensure confidentiality and reduce social desirability bias (e.g., standardized instructions, voluntary participation), future research should consider incorporating multi-source data (e.g., peer or teacher reports) to mitigate this limitation (for a further discussion, see the limitation section).

### Measures

#### Bullying perpetration

Students were asked to reflect on their behavior over the past three months and indicate how often they had engaged in certain actions toward peers who were weaker, less popular, or less powerful than themselves. Importantly, the term "bullying" was not used, and unlike general measures of aggression, this scale explicitly incorporated the concept of power imbalance. The scale included eight items measuring direct bullying, comprising five physical behaviors (e.g., “Hit or kicked someone to cause harm”; “Restrained someone against their will”) and three verbal behaviors (e.g., “Teased or called someone mean names”). Indirect bullying was measured with three items (e.g., “Spread false rumors or lies about someone”). Students responded using a five-point scale ranging from 1 = *never* to 5 = *several times a week*. Cronbach’s α indicated good internal consistency for both subscales (indirect bullying: w1 = 0.83, w2 = 0.75; direct bullying: w1 = 0.90, w2 = 0.85). Mean scores were calculated for each subscale and used in all subsequent analyses.

#### Moral disengagement

Students’ use of moral disengagement mechanisms was assessed using the Moral Disengagement in Bullying Scale (MDBS; [30], which originally comprises 18 items covering all mechanisms defined in Bandura’s framework. Due to technical reasons, unfortunately, one advantageous comparison (see details below) item was omitted from the web-based scale, although all mechanisms were still covered by the instrument. As a result, the MDBS in this study consisted of 17 items. The scale has previously demonstrated good psychometric properties among students in Sweden [3, 30] and in other countries [20, 26, 33]. In the original validation of the scale [30], the mechanisms of dehumanization and blaming the victim were combined into a single factor referred to as victim attribution. Thus, seven moral disengagement mechanisms were measured and analyzed separately in the present study. Each mechanism was assessed by 1 to 4 items, and the following seven composite scales were computed by averaging the items for each mechanism: moral justification, euphemistic labeling, advantageous comparison, displacement of responsibility, diffusion of responsibility, distorting consequences, and victim attribution. Given that moral justification, euphemistic labeling, and diffusion of responsibility were each measured with two items, Spearman-Brown coefficients [7] were reported for these scales, with values ranging from 0.60 to 0.62 (moral justification: w1 = 0.61; euphemistic labeling: w1 = 0.62; diffusion of responsibility: w1 = 0.60), which are slightly below the conventional 0.70 threshold [5]. For displacement of responsibility (w1 = 0.75), distorting consequences (w1 = 0.76), and victim attribution (w1 = 0.77), which were measured with three or more items, Cronbach’s α indicated acceptable to good reliability. Advantageous comparison was measured with a single item since the second item was omitted (i.e., “Teasing a person now and then every week is not so bad if you compare it to hitting and kicking a person every day”) due to technical reasons and was therefore not subject to internal consistency analysis. Mean scores were computed for each mechanism and used in subsequent analyses.

### Statistical analyses

Statistical analyses were conducted in RStudio (version 2025.4.0; [25]. To examine whether moral disengagement mechanisms at Time 1 predicted bullying behavior at Time 2, two linear regression models were specified—one with indirect bullying and one with direct bullying as the dependent variable. A random intercept for school class was included in both models to account for the nested structure of students within classes. This is important because students within the same classroom may be more similar to each other than to students from different classrooms, which can affect the study outcomes. The classroom level has been identified as an essential group context to address in order to better understand and counteract peer victimization (e.g., [13, 15, 28]. The estimated intraclass correlation coefficients (ICCs) indicated that 12% of the variance in direct bullying (between-class variance = 0.011, within-class variance = 0.080) and 11% of the variance in indirect bullying (between-class variance = 0.011, within-class variance = 0.091) was attributable to differences between classes, with the remaining variance residing at the individual level. A likelihood ratio test confirmed that the inclusion of random intercepts for class significantly improved model fit (direct bullying: χ^2^ = 16.61, *p* < 0.001; indirect bullying: χ^2^ = 16.81, *p* < 0.001). Each model was estimated in two steps: Step 1 included gender and prior levels of bullying as control variables, and Step 2 added the moral disengagement mechanisms. In Step 2, we also included interaction effects between gender and moral disengagement mechanisms to explore whether there were gender differences in how these mechanisms predict direct and indirect bullying. All gender interaction terms were tested simultaneously, and we then removed one interaction at a time based on their *p*-values, ending with only the gender by victim attribution interaction for direct bullying being significant. For parsimony, only the significant interaction between gender and victim attribution for direct bullying was retained in the final model. Beyond determining whether the variables of the models were significant, we also evaluated the strength of the significant associations. These effect sizes were computed as ${b}_{k}^{ {\prime}}={b}_{k}* {s}_{xk} / {s}_{y}$; where ${b}_{k}$ is the unstandardized coefficient for variable $k$, ${s}_{xk}$ is the sample standard deviation for the explanatory variable $k$, and ${s}_{y}$ is the sample standard deviation for the dependent variable.

## Results

### Descriptive statistics and correlations

Overall, the levels of both moral disengagement mechanisms and indirect and direct bullying were low. Boys reported significantly higher levels on all study variables except for diffusion of responsibility at Time 1 and indirect bullying at both time points (see Table 1). All bivariate correlations were statistically significant. The moral disengagement mechanisms were moderately to strongly positively interrelated, and each demonstrated significant correlations with both indirect and direct bullying at both time points (see Table 2).

**Table 1:** Means (M), Standard Deviations (SD), and gender comparisons for all study variables

	Total Sample		Girls		Boys		
	*M*	*SD*	*M*	*SD*	*M*	*SD*	*t*
MJ T1	1.95	1.15	1.76	0.93	2.2	1.35	4.42***
EL T1	1.72	1.00	1.59	0.86	1.89	1.14	3.49***
AC T1	1.47	1.05	1.36	0.87	1.62	1.23	2.77**
DIS T1	2.30	1.20	2.18	1.10	2.44	1.3	2.51*
DIF T1	1.65	1.06	1.58	0.99	1.74	1.13	1.77
CON T1	1.54	0.87	1.42	0.70	1.69	1.04	3.49***
VA T1	1.35	0.80	1.24	0.58	1.5	1.0	3.63***
IB T1	1.27	0.51	1.23	0.47	1.32	0.56	1.95
IB T2	1.33	0.50	1.31	0.49	1.35	0.51	1.02
DB T1	1.28	0.46	1.20	0.39	1.37	0.56	4.14***
DB T2	1.29	0.44	1.23	0.41	1.37	0.46	3.51***

*MJ* Moral Justification, *EL* Euphemistic Labeling, *AC* Advantageous Comparison, *DIS* Displacement of Responsibility, *DIF* Diffusion of Responsibility, *CON* Distortion of Consequences, *VA* Victim Attribution, *IB* Indirect Bullying, *DB* Direct Bullying

^*^p < *.05*

^**^p < *.01*

^***^p < *.001*

**Table 2:** Bivariate correlations among study variables

	1	2	3	4	5	6	7	8	9	10	11
1. Moral Justification T1	–										
2. Euphemistic Labeling T1	.44***	–									
3. Advantageous Comparison T1	.43***	.40***	–								
4. Displacement of Responsibility T1	.38***	.43***	.29***	–							
5. Diffusion of Responsibility T1	.32***	.42***	.38***	.52***	–						
6. Distortion of Consequences T1	.47***	.58***	.54***	.55***	.63***	–					
7. Victim Attribution T1	.45***	.50***	.54***	.48***	.58***	.69***	–				
8. Indirect Bullying T1	.27***	.32***	.26***	.23***	.28***	.30***	.40***	–			
9. Direct Bullying T1	.39***	.36***	.32***	.26***	.34***	.40***	.41***	.71***	–		
10. Indirect Bullying T2	.21***	.20***	.15***	.16***	.15***	.12**	.27***	.78***	.57***	–	
11. Direct Bullying T2	.32***	.22***	.18***	.21***	.21***	.22***	.27***	.55***	.74***	.64***	–

^**^p < *.01*

^***^p < *.001*

### Regression analyses

To examine whether moral disengagement mechanisms at Time 1 predicted bullying behavior at Time 2, two linear regression models were estimated—one for direct bullying and one for indirect bullying. All presented model estimates are unstandardized.

#### Direct bullying

**Fig. 1:**
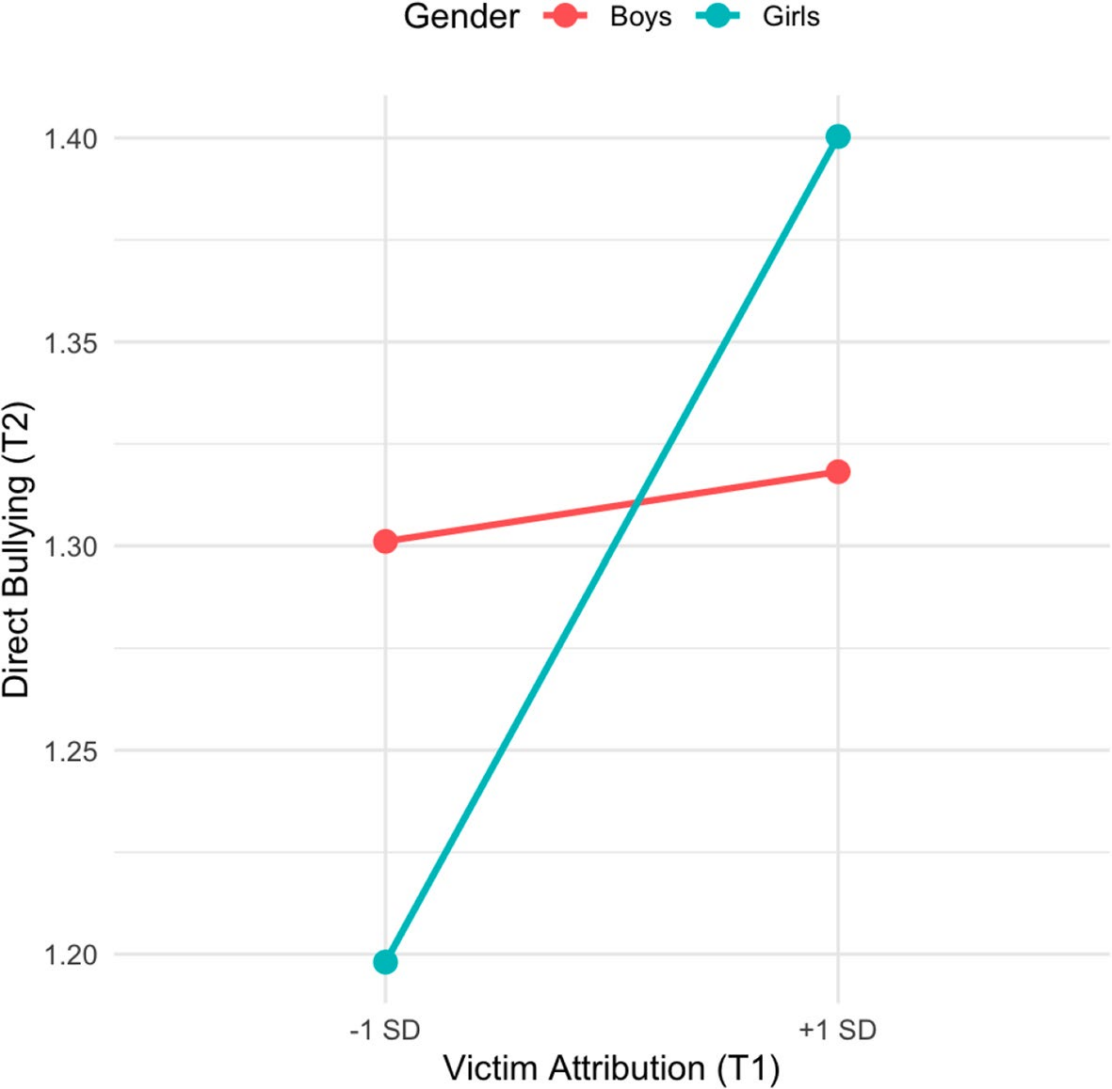
Graphical representation of the interaction between gender and victim attribution on direct bullying

In the direct bullying model (see Table 3), Step 1 showed that prior bullying significantly predicted subsequent bullying (*Est* = 0.73, *p* < 0.001, effect size = 0.75), whereas gender was not a significant predictor (*Est* = –0.02, *p* = 0.42). In Step 2, prior bullying remained a strong predictor (*Est* = 0.73, *p* < 0.001, effect size = 0.75), and gender continued to be non-significant (*Est* = –0.01, *p* = 0.71). In addition, some moral disengagement mechanisms showed small but significant effects. Higher levels of moral justification (*Est* = 0.03, *p* = 0.025, effect size = 0.08) and displacement of responsibility (*Est* = 0.03, *p* = 0.027, effect size = 0.09) at Time 1 were associated with higher levels of direct bullying at Time 2, whereas distortion of consequences (*Est* = –0.05, *p* = 0.039, effect size = –0.10) at Time 1 was associated with lower levels of direct bullying at Time 2. Due to the unexpected negative association for distortion of consequences, we tested for multicollinearity; however, VIFs for all predictors ranged from 1.24 to 2.43, indicating low to moderate multicollinearity. Euphemistic labeling (*Est* = –0.02, *p* = 0.28), advantageous comparison (*Est* = –0.01, *p* = 0.46), and diffusion of responsibility (*Est* = –0.02, *p* = 0.33) were non-significant predictors. The main effect of victim attribution was not statistically significant (*Est* = 0.01, *p* = 0.71); however, a significant interaction with gender (*Est* = 0.12, *p* = 0.004) revealed that victim attribution predicted higher levels of direct bullying among girls only. Follow-up analyses of simple slopes at ± 1 SD of victim attribution confirmed that the effect of victim attribution on direct bullying was significant for girls (*Est* = 0.13, *p* = 0.002), but not for boys (*Est* = 0.01, *p* = 0.708). This interaction is illustrated in Fig. 1, where predicted levels of direct bullying at Time 2 are plotted at −1 and + 1 SD of victim attribution at Time 1, separately for boys and girls.

**Table 3:** Regression estimates for predictors of direct bullying

Predictor	Estimate	*SE*	*p*
Model 1			
Gender	−.025	.028	.37
Direct Bullying T1	.732	.031	<.001
Model 2			
Gender	−.010	.028	.709
Direct Bullying T1	.727	.033	<.001
Moral Justification T1	.033	.015	.023
Euphemistic Labeling T1	−.020	.019	.28
Advantageous Comparison T1	−.013	.018	.46
Displacement of Responsibility T1	.034	.015	.025
Diffusion of Responsibility T1	−.018	.018	.33
Distortion of Consequences T1	−.053	.026	.040
Victim Attribution T1	.010	.029	.73
Gender * Victim Attribution T1	.118	.040	.003

#### Indirect bullying

In the indirect bullying model (see Table 4), Step 1 showed a strong effect of prior bullying (*Est* = 0.76, *p* < 0.001, effect size = 0.77), while gender was not significant (*Est* = 0.02, *p* = 0.43). These patterns remained consistent in Step 2, with prior bullying continuing to predict later bullying behavior (*Est* = 0.76, *p* < 0.001, effect size = 0.76), and gender remaining non-significant (*Est* = 0.04, *p* = 0.23). Additionally, some moral disengagement mechanisms showed small but significant effects. Moral justification (*Est* = 0.03, *p* = 0.031, effect size = 0.07), displacement of responsibility (*Est* = 0.05, *p* = 0.004, effect size = 0.10), and victim attribution (*Est* = 0.07, *p* = 0.022, effect size = 0.10) were each associated with higher levels of indirect bullying. In contrast, distortion of consequences (*Est* = –0.08, *p* = 0.005, effect size = –0.13) was linked to lower levels of indirect bullying. Due to the unexpected negative association for distortion of consequences, we tested for multicollinearity; however, VIFs for all predictors ranged from 1.13 to 2.43, indicating low to moderate multicollinearity. Euphemistic labeling (*Est* = –0.02, *p* = 0.32), advantageous comparison (*Est* = 0.00, *p* = 0.96), and diffusion of responsibility (*Est* = –0.03, *p* = 0.21) were non-significant predictors.

**Table 4:** Regression estimates for predictors of indirect bullying

Predictor	Estimate	*SE*	*p*
Model 1			
Gender	.023	.030	.43
Indirect Bullying T1	.780	.029	<.001
Model 2			
Gender	.035	.030	.23
Indirect Bullying T1	.765	.031	<.001
Moral Justification T1	.033	.015	.031
Euphemistic Labeling T1	−.020	.020	.32
Advantageous Comparison T1	.001	.019	.96
Displacement of Responsibility T1	.045	.016	.004
Diffusion of Responsibility T1	−.025	.020	.21
Distortion of Consequences T1	−.077	.027	.005
Victim Attribution T1	.067	.029	.022

## Discussion

The current study investigated whether specific mechanisms of moral disengagement predict subsequent bullying perpetration among upper elementary school students in Sweden. Drawing on Bandura’s [2] conceptualization of moral disengagement as a multidimensional construct, the study examined whether each mechanism predicted direct and indirect bullying over time. The study hypothesized that both moral justification and victim attribution would be positively associated with subsequent bullying perpetration Utilizing data from two measurement points within the same academic year, and controlling for gender and baseline levels of bullying, the results revealed both converging and diverging patterns across the two forms of bullying.

In line with our hypotheses, moral justification was consistently associated with higher levels of both direct and indirect bullying. This finding aligns with previous research suggesting that cognitively reframing harmful behavior as morally defensible—for example, as necessary or justified in response to the victim’s behavior—can reduce moral restraint and facilitate aggressive behavior over time [3, 19, 30]. These findings further support the notion that the behavioral locus of moral disengagement is associated with bullying involvement over time (also see [12], particularly through moral justification as a key cognitive strategy.

Victim attribution also emerged as a relevant mechanism, though its association with bullying varied across contexts. Specifically, it predicted higher levels of indirect bullying overall and was significantly associated with direct bullying only among girls. One possible explanation is that direct forms of aggression, such as verbal or physical attacks, are less socially acceptable for girls [8], who may therefore require stronger cognitive justifications to engage in such behavior (see also [3]. Victim-blaming may thus serve as a particularly salient mechanism for bypassing moral self-sanctions among girls when they engage in overt bullying. This pattern is consistent with findings from Bjärehed et al. [3], who likewise reported a significant interaction between gender and victim attribution in predicting direct bullying, but not indirect bullying. In their study, however, the main effect of victim attribution on direct bullying also remained significant, suggesting that this mechanism can also play a broader role in direct bullying, although potentially amplified among girls. Taken together, these findings highlight the gendered dynamics of moral reasoning in bullying situations and suggest that victim attribution may be especially important to address in interventions targeting direct aggression among girls.

Beyond these two moral disengagement mechanisms, displacement of responsibility and distortion of consequences also emerged as significant predictors of both direct and indirect bullying. Displacement of responsibility enables aggressors to minimize personal accountability by attributing responsibility to others—such as peers, group norms, or authority figures—and may thereby reduce internal moral conflict and facilitate repeated engagement in bullying. While previous studies have consistently reported positive bivariate associations between this mechanism and bullying perpetration (e.g., [3, 30], to our knowledge, no prior research has demonstrated a unique predictive effect in a multivariate model. The current findings therefore provide preliminary evidence that displacement of responsibility may account for variance in bullying behavior beyond other moral disengagement mechanisms. In his seminal work, Olweus [21] distinguishes between bullies who initiate bullying and assume a leadership role and those who participate in bullying perpetration without initiating the behavior or occupying a leading position. The latter are referred to as “henchmen” or “followers” (see also [22]. This distinction aligns with the participant role approach to school bullying, which differentiates between “bullies” and “assistants” [27]. It is plausible that displacement of responsibility operates differently across these two bully roles, as those who initiate and lead bullying may have different motivations and strategies compared to those who follow or assist without initiating the behavior. Future research should therefore investigate whether “henchmen”/“followers” or “assistants” are more likely than those who initiate and lead bullying to attribute responsibility for the bullying to leaders, peer groups, or other figures of power, status, or authority.

Interestingly, distortion of consequences was negatively associated with both direct and indirect bullying in the multivariate models. This finding stands in contrast to theoretical expectations and previous cross-sectional studies, where this mechanism has typically shown positive associations with bullying perpetration (e.g., [3, 11]. One possible explanation is that this is a result of a suppressor effect: as seen in the bivariate correlations, distortion of consequences was significantly and positively related to bullying outcomes, but also showed the strongest intercorrelations with all other moral disengagement mechanisms. It is therefore plausible that when statistically controlling for these overlapping mechanisms, particularly victim attribution and moral justification, a distinct and opposite association emerges. In other words, students who do not attempt to minimize or deny the harm they cause (i.e., score low on this mechanism), but who do score high on other disengagement mechanisms, may be more likely to engage in bullying. One hypothesis for future research is that there may be a subgroup of students who disengage morally through other justifications (e.g., blaming the victim or justifying their behavior) yet remain fully aware of the harm they inflict. This finding may be interpreted as supporting the original definition of bullying, which emphasizes the intention to inflict harm [17, 21], or as indicating that individuals who engage in bullying are, at a minimum, aware that their behavior results in the victim’s distress or suffering. This is also supported by findings on affective and cognitive empathy, which suggest that bullying perpetrators tend to display lower levels of cognitive and, in particular, affective empathy (for a review, see [34]. Future research could explore whether distortion of consequences functions differently depending on the presence or strength of other mechanisms, for example, through interaction analyses or person-centered approaches such as latent profile analysis. Moreover, longitudinal studies that assess changes in moral disengagement mechanisms over time could help determine whether distortion of consequences acts as an early warning signal or instead emerges later in the process as a cognitive adjustment after engaging in harmful behavior.

### Limitations and suggestions for future research

Despite the strengths of this study, several limitations should be acknowledged. First, the reliance on self-report measures may introduce biases related to perception, recall, social desirability, and underreporting [23], particularly given the sensitive nature of moral disengagement and bullying behaviors. Although efforts were made to ensure confidentiality, it is possible that some students minimized or exaggerated their responses. Moreover, the use of self-report measures may have inflated associations among variables due to shared method variance. To further validate and extend these findings, future research should employ multisource data, such as peer nomination procedures, to assess bullying perpetration.

Second, while the longitudinal design strengthens the ability to infer temporal relationships, the study was limited to two measurement points within a single academic year. This relatively short interval constrains the ability to examine longer-term developmental trajectories and may also partly explain the small effect sizes observed, as the high stability of bullying behaviors over the two time points and the control for prior levels of bullying reduced the available variance for detecting new effects.

Third, due to technical reasons, one item from the Moral Disengagement in Bullying Scale (MDBS) was omitted. While all mechanisms were still covered and the scale demonstrated acceptable reliability, the absence of this item may have affected the measurement precision, particularly for the advantageous comparison mechanism, which was assessed with a single item. Single-item measures are inherently limited in their ability to capture the full breadth of a construct and may introduce additional measurement error. Furthermore, some scales were measured with two items (i.e., moral justification, euphemistic labeling, and diffusion of responsibility) and displayed relatively low reliability (≈0.60), which may have reduced measurement precision and attenuated effect sizes. Future research should prioritize the use of multi-item scales with higher reliability to improve measurement precision and better capture the constructs of interest.

Fourth, the study’s analytical approach controlled for gender and prior bullying behavior, but did not include other factors that have been shown to be relevant in previous research, such as empathy [34], callous-unemotional traits [38], class climate andteacher practices [31], or peer network characteristics [35], which could also influence bullying behavior and moral disengagement. Future research should explore how such factors may moderate the association between moral disengagement and bullying perpetration.

Fifth, although attrition analyses indicated no significant differences at Time 1 between students included in the longitudinal analyses and those excluded due to missing data, attrition remains a potential source of bias. Students who did not participate at both time points may differ in unmeasured ways that could influence the results. Finally, while the sample included students from a variety of socioeconomic and geographic backgrounds within Sweden, it was restricted to Swedish upper elementary schools. This potentially limits the generalizability of the findings to other cultural contexts, as norms around aggression, morality, and gender may differ across cultures. Cross-cultural replications are therefore needed to determine the generalizability of these findings.

### Implications for practice

The findings have several important implications for anti-bullying interventions in schools. First, the results underscore the need to address specific moral disengagement mechanisms—particularly moral justification, displacement of responsibility, and victim attribution—within prevention efforts. Although the effect sizes were small, they are meaningful in the context of bullying prevention as even small shifts in students' cognitive justifications for harmful behavior can have cumulative or long-term impacts on bullying dynamics in classrooms. As our study suggests, moral justification was a significant predictor of direct and indirect bullying, as was victim attribution to some degree.It is also imperative that school practitioners in Sweden closely assess students’ motives for bullying others. For those who felt that it was morally justified to bully others, practitioners might consider intervention programs that target students’ sense of morals. Moreover, such cognitive processes may serve as early warning signals, and identifying and intervening on disengagement patterns before behavioral problems escalate could be a fruitful avenue for both universal and targeted prevention efforts. Programs, such as the Moral Reconation Therapy, an evidence-based counseling program, combine education, counseling, and structured exercises to foster moral development. MRT has shown promise in reducing problematic behaviors, such as substance use and delinquency (e.g., [10]). More importantly, programs such as MRT that aim to foster moral engagement should not only teach students that bullying is wrong but also explicitly challenge the specific rationalizations they may use to justify such behavior.

Also, our finding suggests that victim attribution predicted a higher level of indirect bullying, and it was a significant predictor among girls, which suggests that girls may bully those directly and indirectly (e.g., relational aggression) whom they perceive as “deserving it”. The identified gender interaction suggests that interventions may benefit from being sensitive to the different ways in which boys and girls morally rationalize aggression. In particular, discussions around victim blaming may be particularly relevant for addressing direct bullying among girls.

## Data Availability

The datasets generated and/or analyzed during the current study are not publicly available due to ethical and privacy restrictions but are available from the corresponding author on reasonable request.
